# Supplementary material for: Unveiling the role of DHIS2 in enhancing data quality and accessibility in primary healthcare facilities: Evidence from Ethiopia
Source: PLoS One. 2024 Dec 18;19(12):e0314505. doi: 10.1371/journal.pone.0314505 (PMC11654967; doi:10.1371/journal.pone.0314505)
Supplement: Attachment — Submitted filename: supporting file for plos review invitation.pdf [file pone.0314505.s001.pdf]

Review Report

The manuscript titled " Unveiling DHIS2's Impact on Enhancing Data Quality, Availability, and Performance of PHU Facilities: Insights from Ethiopia" investigates the impact influencing the data quality, availability and performance in Ethiopia. The study is timely and relevant, addressing the increasing importance of digital health technologies in resource-limited settings. The manuscript addresses an important and timely topic with a well-structured study design and robust frameworks. However, to enhance the clarity, depth, and impact of the study, the authors should consider the revisions and recommendations outlined below. This will help ensure the findings are both comprehensible and actionable for a broader audience.

Strengths

The topic is highly relevant, given the global push towards digital health solutions, particularly in low-resource settings. The use of the qualitative studies by selecting four cities including Addis Ababa it sounds a robust theoretical framework for understanding the DHIS2's Impact on Enhancing Data Quality, Availability, and Performance of PHU Facilities. The study employs a well-defined sampling technique and uses ATLAS.ti software for data analysis. Ethical approval and participant consent were obtained, ensuring the study adheres to ethical research standards.

Limitations and Areas for Improvement

The study lacks practical implications and the author should be including the strength and limitations that ware faced in the provided study.

Language and Clarity

There are some areas where the language could be clearer and more concise. Review the manuscript for clarity and conciseness. Ensure that technical terms are well-defined and that the narrative flow is logical and easy to follow.

Practical Implications

The practical implications of the findings could be more explicitly stated. The authors should emphasize how the findings can inform the impact on enhancing data quality,

availability and performance DHIS2 systems in Ethiopia. Provide specific recommendations for healthcare providers, policymakers, and technology developers

Specific Comments

Abstract

Ensure the abstract succinctly summarizes the key findings and their significance. Consider including specific recommendations derived from the study.

Introduction

Provide more context on the DHIS2's Impact on Enhancing Data Quality, Availability, and Performance of PHU Facilities in Ethiopia. Highlight the gap in knowledge regarding DHIS2 adoption in low-resource settings

Literature Review

Expand the literature review to include more studies on DHIS2 related to data quality, availability and performance globally and in similar contexts. This will help position the study within the broader research landscape

Results

Should be write clearly presented with appropriate saturated levels.

Discussion

Discussion should be rewrite succinctly. To make suitable and make easy for comments towards the reviewer the author should use continuous line number for all of your manuscript.

For example from the discussion part the auther try to write like the following ….. phrase

"Studies conducted in [insert country name] have reported similar outcomes, emphasizing DHIS2's effectiveness in improving data quality and management processes. These comparisons underscore the global recognition of DHIS2 as a valuable tool for strengthening health information systems and driving positive change in healthcare delivery worldwide."
