# Supplementary material for: Unveiling the role of DHIS2 in enhancing data quality and accessibility in primary healthcare facilities: Evidence from Ethiopia
Source: PLoS One. 2024 Dec 18;19(12):e0314505. doi: 10.1371/journal.pone.0314505 (PMC11654967; doi:10.1371/journal.pone.0314505)
Supplement: Attachment — Submitted filename: DHIS2 plose.docx [file pone.0314505.s002.docx]

This the points the researcher considered

1. I am positive this paper is published
2. The problem is clearly mentioned
3. The sampling procedure, convenience and purposive sampling strategies were utilized to identify and recruit participants for key informant interviews and focus group discussions. Explain separate for which convenience sampling? KII or FGD and purposive sampling?
4. In sample size and Sampling Technique section said that a total of 30 KIIs and 16 FGDs were conducted. Not clear, what does it mean how money participants were participate in 16 FGD?
